# Inverse relationship between serum haptoglobin and acute kidney injury in critically Ill patients with sepsis: A retrospective cohort study of the MIMIC-IV 3.0 database

**DOI:** 10.1016/j.clinsp.2025.100725

**Published:** 2025-07-30

**Authors:** Yue Liao, Qilin Yang, Daxue Li, Yulong Wang

**Affiliations:** a Department of Pharmacy, Chongqing General Hospital, Chongqing University, Chongqing, China; b Department of Critical Care Medicine, The Second Affiliated Hospital of Guangzhou Medical University, Guangdong Province, China; c Department of Breast and Thyroid Surgery, Chongqing Health Center for Women and Children, Women and Children's Hospital of Chongqing Medical University, Chongqing, China; d Department of Ophthalmology, Chongqing General Hospital, Chongqing University, Chongqing, China

**Keywords:** Haptoglobins, Acute kidney injury, Sepsis, Hemoglobins, MIMIC-IV

## Abstract

**Background:**

Previous studies have indicated that perioperative serum haptoglobin levels are associated with the risk of Acute Kidney Injury (AKI). However, the relationship between haptoglobin and AKI in sepsis patients remains unclear. This study aimed to investigate the association between serum haptoglobin levels and AKI in critically ill sepsis patients.

**Methods:**

The authors conducted a retrospective cohort study using data extracted from the Medical Information Mart for the Intensive Care (MIMIC) IV database version 3.0. Multivariable logistic regression analysis was used to control for potential confounders. Curve fitting was performed to assess the linearity of the association, and subgroup analyses were conducted to validate the robustness of the findings.

**Results:**

This study included 1324 participants (mean age 64.7 ± 17.6 years; 53.8 % male). The fully adjusted model showed an inverse association between serum haptoglobin levels and AKI. Each 10 mg/dL increase in haptoglobin decreased AKI odds by 1.4 % (OR = 0.986, 95 % CI 0.973‒0.999, *p* = 0.037). Patients in the highest haptoglobin group had 34.5 % lower AKI risk versus the lowest group (OR = 0.655, 95 % CI 0.439‒0.976, *p* = 0.038). The linear relationship (p for non-linearity = 0.152) remained robust in subgroup analyses.

**Conclusion:**

The present findings suggest that elevated serum haptoglobin levels may serve as a protective factor against AKI in critically ill sepsis patients. Nevertheless, prospective, randomized, controlled studies are required to confirm the findings presented in this investigation.

## Highlights

• Identified an inverse association between serum haptoglobin levels and sepsis-associated AKI risk.
• First evidence of haptoglobin as a predictive biomarker for AKI in septic ICU patients.
• Highlights haptoglobin's potential for AKI prevention and intervention in sepsis.

## Introduction

Sepsis, characterized by the host's detrimental response to infection, is a leading cause of organ dysfunction^1^ and impacts millions of individuals globally annually, contributing to nearly one in five of all deaths worldwide.^2^^,^^3^ It has emerged as a critical public health challenge. Sepsis-Associated Acute Kidney Injury (SA-AKI) represents the most prevalent organ dysfunction and is independently linked to higher mortality rates.^4^ In the context of sepsis, multiple pathways can precipitate Acute Kidney Injury (AKI).^5^ Particularly, elevated free hemoglobin levels exert a profound impact on the pathogenesis of renal impairment, this influencing this through a variety of mechanisms, including oxidative stress, tubular obstruction, consumption of nitric oxide, and immune dysregulation.^6^, ^7^, ^8^ Considering the absence of effective measures to prevent or manage AKI in sepsis, identifying potential therapeutic interventions is crucial.

Haptoglobin is a member of the acute-phase plasma protein family and serves as the primary plasma detoxifier of hemoglobin.^9^ During hemolysis, it tightly captures free hemoglobin, forming complexes that reduce the oxidative potential of heme and facilitate recognition and phagocytosis by the macrophage scavenger receptor CD163.^10^ Despite continuous synthesis by the liver, the capacity of the haptoglobin-hemoglobin clearance system is overwhelmed when the rate of free hemoglobin release is high.^11^^,^^12^ Previous studies have shown that lower haptoglobin levels in septic patients are associated with higher mortality rates^13^^,^^14^ and that admission haptoglobin concentrations have been shown to predict AKI in patients undergoing cardiac surgery, as well as those with Acute Respiratory Distress Syndrome(ARDS) or severe burns.^15^, ^16^, ^17^, ^18^ However, research on its correlation with sepsis-associated AKI is still scarce.

In the present study, the authors aimed to assess the association between early haptoglobin levels upon Intensive Care Unit (ICU) admission and the subsequent development of AKI in septic patients. The authors sought to determine the optimal range of haptoglobin levels early in septic patients and explore its potential as a therapeutic target for improving sepsis outcomes.

## Materials and methods

The authors obtained the data from the Medical Information Mart for the Intensive Care Database IV (MIMIC-IV) database version 3.0.^19^ The database comprises data from over 90,000 patients admitted to Beth Israel Deaconess Medical Center's ICUs in Boston, MA, from 2008 to 2022. The ethical approval statement and informed consent were not required for this study because all personal information was replaced with random codes and anonymized. Author Yue Liao completed a training programme facilitated by the PhysioNet team and secured official approval to use the MIMIC-IV database (certification ID: 60,723,603). The study's methodology and reporting adhered to the guidelines set forth by the Strengthening the Reporting of Observational Studies in Epidemiology (STROBE) initiative.^20^

### Study population

The study population consisted of individuals with sepsis and subsequently admitted to the ICU. The diagnosis of sepsis was established through the Third International Consensus Definitions for Sepsis and Septic Shock (Sepsis-3) definitions.^1^ Exclusion criteria were defined as follows: 1) Patients with sepsis admitted to the ICU not for the first time; 2) ICU length of stay <48 h; 3) The age of participants under 18 years old; 4) Missing serum haptoglobin data; 5) Patients with thalassemia.

### Definitions

According to the Kidney Disease Improving Global Outcomes (KDIGO) guidelines,^21^ AKI is defined as a serum creatinine increase of ≥0.3 mg/dL within 48 h post-admission to the ICU. Serum haptoglobin was collected from the first measurement taken following ICU admission within 24 h.

### Demographical and laboratory variables

The authors queried patient information from the MIMIC-IV database using Structured Query Language (SQL), and all covariates were collected on the first day of ICU admission. Data on the following were extracted: 1) Demographic variables, including age, sex; 2) Vital signs, including Systolic Blood Pressure (SBP), Diastolic Blood Pressure (SBP), body temperature, respiratory rate; 3) Comorbidities, including hypertension, diabetes, chronic pulmonary disease, myocardial infarction; 4) Laboratory parameters, including White Blood Cell Count (WBC), platelet count, glucose, Aminotransferase (ALT), Aspartate Aminotransferase (AST), bilirubin, Blood Urea Nitrogen (BUN), blood creatinine, sodium, potassium, chloride; 5) Illness severity scores, including Sequential Organ Failure Assessment (SOFA) score and Acute Physiology Score III (APSIII). If repeated test results presented themselves within 24 h, only the worst value was selected.

### Outcomes

The outcome measured was early AKI, which was identified within 2 days after ICU admission.

### Statistics

Serum haptoglobin levels were classified into three groups. Continuous variables were described using mean ± Standard Deviation (SD) or median Interquartile Range (IQR), while categorical variables were presented as percentages. *T*-tests or Wilcoxon rank-sum tests were used for continuous variables, and Chi-Squared tests were used for categorical variables.

The association between serum haptoglobin levels and AKI was investigated using univariate and multivariable logistic regression models. Three models were used: Model 1 was adjusted for age, sex; Model 2 was adjusted for age, sex, SBP, DBP, temperature, platelets, BUN, creatinine, sodium, potassium, chloride, AST, bilirubin, diabetes, APSIII, SOFA; Model 3 was adjusted for age, sex, SBP, DBP, respiratory rate, temperature, WBC, platelets, glucose, BUN, creatinine, sodium, potassium, chloride, ALT, AST, bilirubin, hypertension, diabetes, chronic pulmonary disease, APSIII and SOFA.

A restricted cubic spline was applied to investigate the linear relationship between serum haptoglobin and AKI in patients with sepsis. Additionally, the authors conducted subgroups and interaction analyses to assess the underlying clinical heterogeneity.

The percentages of covariates with missing data are presented in Supplementary Table 1. All variables had < 10 % missingness, so the authors conducted an analysis of patients after excluding those with missing data from the study. All statistical analyses were conducted using R 4.2.2 (http://www.R-project.org, R Foundation) and Free Statistics software version 1.9.1; *p* < 0.05 was considered statistical significance.

## Results

### Baseline characteristics and clinical characteristics

**Fig. 1:**
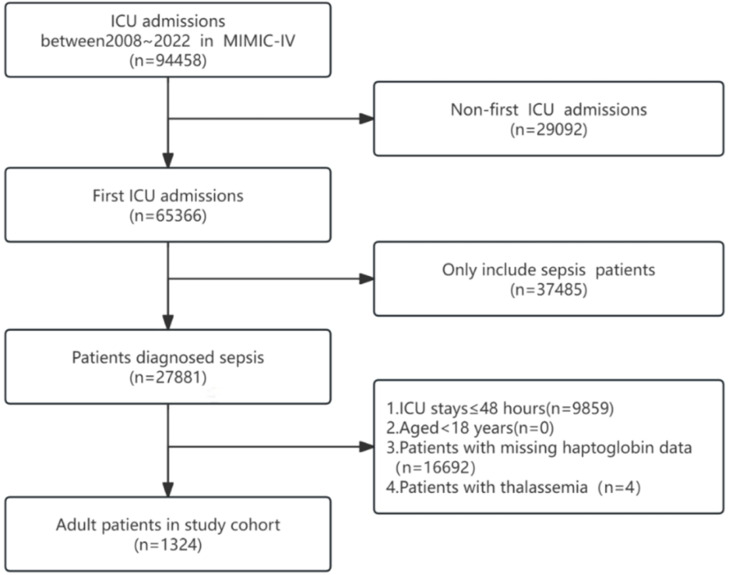
Flow chart of study population.

Following the screening process, a total of 1324 patients met the inclusion criteria for this study, as illustrated in the flowchart presented in Fig. 1. Baseline characteristics of the patients are shown in Table 1. The average age was 64.0 ± 17.6 years, and the majority were males (53.8 %; *n* = 712). In accordance with serum haptoglobin levels stratified by three groups, individuals with higher serum haptoglobin levels tend to have higher Systolic Blood Pressure (SBP), faster respiratory rates, and increased platelet counts and blood glucose, while also exhibiting lower levels of Alanine Transaminase (ALT), Aspartate Transaminase (AST), total bilirubin, and lower SOFA scores.

**Table 1:** Baseline characteristics of participants grouped according to haptoglobin levels.

Variables	Total (*n* = 1324)	Group 1 (*n* = 455)	Group 2 (*n* = 396)	Group 3 (*n* = 473)	p-value
		< 100 (mg/mL)	100∼200 (mg/mL)	≥200 (mg/mL)	
Gender, n ( %)					0.466
Female	612 (46.2)	200 (44)	190 (48)	222 (46.9)	
Male	712 (53.8)	255 (56)	206 (52)	251 (53.1)	
Age(years)	64.0 ± 17.6	62.5 ± 17.6	64.6 ± 18.7	65.0 ± 16.5	0.07
SBP (mm Hg)	113.7 ± 16.2	111.9 ± 15.6	113.9 ± 16.6	115.3 ± 16.2	0.005
DBP (mm Hg)	62.4 ± 11.2	62.5 ± 11.3	62.5 ± 11.4	62.3 ± 10.9	0.943
Respiratory rate (breaths/min)	21.5 ± 4.6	20.9 ± 4.5	21.1 ± 4.4	22.4 ± 4.7	<0.001
Temperature ( °C)	37.6 ± 0.9	37.6 ± 0.9	37.5 ± 0.8	37.7 ± 0.9	<0.001
WBC (×10^9^/L)	13.7 (8.8, 19.6)	12.7 (8.1, 19.5)	13.4 (8.8, 19.5)	14.3 (9.2, 19.8)	0.163
Platelets (×10^12^/L)	127.5 (67.8, 208.0)	95.0 (51.0, 143.0)	133.5 (75.0, 201.0)	177.0 (100.0, 280.0)	<0.001
Glucose (mg/mL)	114.4 ± 40.8	109.4 ± 39.2	113.9 ± 41.2	119.7 ± 41.5	<0.001
ALT (IU/L)	36.0 (19.0, 102.2)	44.0 (23.0, 148.0)	34.0 (18.0, 86.0)	32.0 (18.0, 75.0)	<0.001
AST (IU/L)	60.0 (30.0, 187.0)	95.0 (39.0, 282.0)	54.0 (28.0, 161.5)	45.0 (26.0, 109.0)	<0.001
Bilirubin total (mg/mL)	1.0 (0.5, 2.4)	1.7 (0.8, 4.4)	0.9 (0.4, 2.1)	0.7 (0.4, 1.2)	<0.001
BUN (mg/mL)	33.0 (20.0, 56.0)	31.0 (19.0, 50.0)	34.0 (21.0, 57.0)	35.0 (21.0, 59.0)	0.093
Creatinine (mg/mL)	1.6 (1.0, 2.8)	1.6 (1.0, 2.8)	1.6 (1.0, 2.7)	1.5 (0.9, 3.0)	0.292
Sodium(mmoL/L)	139.5 ± 6.0	139.3 ± 6.2	139.6 ± 5.5	139.6 ± 6.1	0.735
Potassium (mmoL/L)	4.7 ± 1.0	4.8 ± 1.0	4.8 ± 1.0	4.7 ± 0.9	0.136
Chloride (mmoL/L)	105.3 ± 7.3	105.3 ± 7.6	105.5 ± 7.2	105.1 ± 7.2	0.699
Hypertension, n ( %)	405 (30.6)	141 (31)	107 (27)	157 (33.2)	0.141
Diabetes, n ( %)	401 (30.3)	129 (28.4)	126 (31.8)	146 (30.9)	0.516
Chronic Pulmonary Disease, n ( %)	341 (25.8)	102 (22.4)	102 (25.8)	137 (29)	0.074
Myocardial infarct, n ( %)	288 (21.8)	94 (20.7)	89 (22.5)	105 (22.2)	0.78
APSIII score	60.3 ± 23.7	63.6 ± 24.5	58.0 ± 22.3	58.9 ± 23.8	<0.001
SOFA score	3.8 ± 2.6	4.2 ± 2.9	3.7 ± 2.5	3.5 ± 2.3	<0.001
AKI, n ( %)	1028 (77.6)	375 (82.4)	307 (77.5)	346 (73.2)	0.003
AKI stage, n ( %)					0.001
1	271 (20.5)	96 (21.1)	80 (20.2)	95 (20.1)	
2	428 (32.3)	137 (30.1)	138 (34.8)	153 (32.3)	
3	329 (24.8)	142 (31.2)	89 (22.5)	98 (20.7)	

SBP, Systolic Blood Pressure; DBP, Diastolic Blood Pressure; WBC, White Blood Cell; ALT, Alanine Aminotransferase; AST, Aspartate Aminotransferase; BUN, Blood Urea Nitrogen; APSIII, Acute Physiology Score III; SOFA, Sequential Organ Failure Assessment.

### Associations between haptoglobin and AKI

When haptoglobin was considered as a continuous variable, the univariate logistic regression analysis (Table 2) revealed a significant inverse relationship between haptoglobin levels and the occurrence of AKI (OR = 0.98, 95 % CI 0.97‒0.99, *p* < 0.001). This association remained statistically significant after adjusting for potential confounders in the multivariate analysis (Table 3), with higher serum haptoglobin levels associated with a reduced risk of AKI in sepsis patients. Specifically, each 10 mg/dL increase in serum haptoglobin levels corresponded to a 1.4 % lower odds of AKI (OR = 0.986, 95 % CI 0.973‒0.999, *p* = 0.037). Furthermore, when haptoglobin was categorized into quartiles and adjusted for various factors including age, sex, SBP, DBP, respiratory rate, temperature, WBC, platelets, glucose, bun, creatinine, sodium, potassium, chloride, ALT, AST, bilirubin, hypertension, diabetes, chronic pulmonary disease, APSIII and sofa score, a notable trend was observed (p for trend < 0.05), compared to the lowest serum haptoglobin level group (< 100 mg/dL), the risk of AKI in the highest serum haptoglobin level group (> 200 mg/dL) was reduced by 34.5 % (OR = 0.655, 95 % CI 0.439‒0.976, *p* = 0.038) (Table 3).

**Table 2:** Univariate logistic regression analysis of incidence of acute kidney injury.

Variable	OR (95 % CI)	p-value
Gender (Male vs. female)	1.04 (0.8∼1.35)	0.773
Age	1.02 (1.01∼1.02)	<0.001
SBP (mm Hg)	0.99 (0.98∼1)	0.027
DBP (mm Hg)	0.98 (0.97∼0.99)	0.001
Respiratory rate (breaths/min)	1.01 (0.98∼1.04)	0.585
Temperature ( °C)	0.83 (0.72∼0.96)	0.013
WBC (×10^9^/L)	1.01 (1∼1.02)	0.166
Platelets (×10^12^/L)^a^	0.81 (0.7∼0.94)	0.007
Haptoglobin (per 10mg/dL)	0.98 (0.97∼0.99)	<0.001
Glucose (mg/mL)^a^	1.12 (0.77∼1.61)	0.562
BUN (mg/mL)	1.01 (1.01∼1.02)	<0.001
Creatinine (mg/mL)	1.47 (1.32∼1.65)	<0.001
Sodium(mmoL/L)	0.97 (0.95∼1)	0.021
Potassium (mmoL/L)	1.44 (1.23∼1.69)	<0.001
Chloride (mmoL/L)	0.98 (0.96∼0.99)	0.01
ALT (IU/L)^a^	1.15 (1.04∼1.26)	0.007
AST (IU/L)^a^	1.27 (1.15∼1.41)	<0.001
Bilirubin total (mg/mL)	1.06 (1.02∼1.11)	0.006
Hypertension (Yes vs. No)	0.91 (0.69∼1.21)	0.524
Diabetes (Yes vs. No)	1.61 (1.19∼2.17)	0.002
Chronic Pulmonary Disease (Yes vs. No)	1.13 (0.84∼1.52)	0.43
APSIII score	1.03 (1.03∼1.04)	<0.001
SOFA score	1.13 (1.07∼1.2)	<0.001

a To normalize the distribution of variable, which exhibited a highly skewed distribution, the authors applied a natural logarithm transformation. OR, Odds Ratio; CI, Confidence Interval.

**Table 3:** Multivariable logistic regression to assess the association of haptoglobin with acute kidney injury.

Variable	Unadjusted		Model 1		Model 2		Model 3	
	OR (95 % CI)	p-value	OR (95 % CI)	p-value	OR (95 % CI)	p-value	OR (95 % CI)	p-value
Haptoglobin (per 10 mg/dL)	0.98 (0.971∼0.99)	<0.001	0.979 (0.969∼0.989)	<0.001	0.985 (0.972∼0.998)	0.024	0.986 (0.973∼0.999)	0.037
Haptoglobin Group, mg/dL								
< 100	1 (Ref)		1 (Ref)		1 (Ref)		1 (Ref)	
≥ 100, < 200	0.736 (0.525∼1.031)	0.075	0.707 (0.502∼0.994)	0.046	0.879 (0.595∼1.299)	0.519	0.835 (0.562∼1.242)	0.374
≥ 200	0.581 (0.424∼0.797)	<0.001	0.547 (0.398∼0.754)	<0.001	0.683 (0.465∼1.005)	0.053	0.655 (0.439∼0.976)	0.038
p for trend. test		<0.001		<0.001		0.05		0.037

Model 1 = Adjust for (age + sex).

Model 2 = Model 1 + SBP + DBP + temperature + platelets + BUN + creatinine + sodium + potassium + chloride + AST + bilirubin + diabetes + APSIII + SOFA.

Model 3 = Model 2 +respiratory rate + WBC + glucose + hypertension + chronic pulmonary disease.

**Fig. 2:**
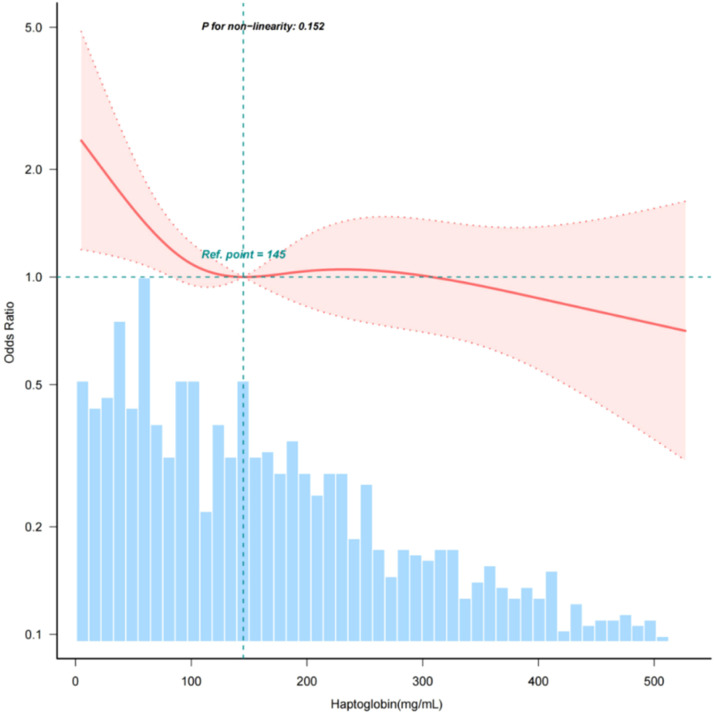
The relationship between haptoglobin and acute kidney injury of patients with sepsis. Adjustment factors included gender, age, SBP, DBP, respiratory rate, temperature, WBC, platelets, glucose, BUN, creatinine, sodium, potassium, chloride, ALT, AST, bilirubin, hypertension, diabetes, chronic pulmonary disease, APSIII, SOFA.

In order to assess the linear correlation between serum haptoglobin and AKI in sepsis patients, the authors utilized a smooth curve fitting approach. Following adjustment for confounding factors, a statistically significant linear association was observed between serum haptoglobin levels and AKI (p fornon-linearity > 0.05) (Fig. 2).

### Subgroup analysis

**Fig. 3:**
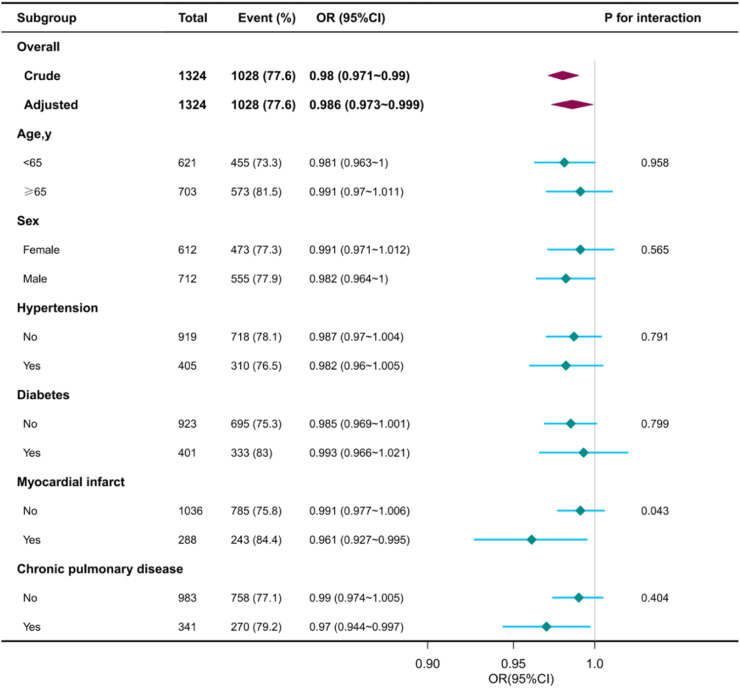
Subgroup analyses of the serum haptoglobin and acute kidney injury. Except for the stratification component itself, each stratification factor was adjusted for gender, age, SBP, DBP, respiratory rate, temperature, WBC, platelets, glucose, BUN, creatinine, sodium, potassium, chloride, ALT, AST, bilirubin, hypertension, diabetes, chronic pulmonary disease, APSIII, SOFA.

The authors conducted subgroup analyses to evaluate potential effect modifications in the association between serum haptoglobin and AKI. Participants were categorized based on age, sex, hypertension (yes or no), diabetes (yes or no), myocardial infarction (yes or no), and chronic obstructive pulmonary disease (yes or no) (Fig. 3). The present study results showed that the relationship remained robust and reliable. However, there was an interaction observed among patients with myocardial infarction (p-value < 0.05).

## Discussion

In this retrospective cohort study, the authors observed a significant inverse linear association between serum haptoglobin levels and AKI in septic patients, with similar results observed across different subgroups. The present study reveals that in comparison with sepsis patients who had serum haptoglobin levels below 100 mg/dL upon ICU admission, those with levels exceeding 200 mg/dL exhibited a 34.5 % decrease in the risk of AKI (OR = 0.655, 95 % CI 0.439‒0.976, *p* = 0.038).

Consistent with these findings, previous studies have indicated that lower serum haptoglobin levels are associated with an increased risk of AKI in patients. For instance, Mamikonian et al. conducted a prospective observational study on 40 pediatric patients (aged 3 days to 4.8 years) undergoing cardiac surgery with Cardiopulmonary Bypass (CPB) and found that pediatric cardiopulmonary bypass induces significant hemolysis, persistently elevated levels of plasma hemoglobin were associated with a fivefold increase in the risk of AKI. The increase in plasma hemoglobin levels, accompanied by a decrease in haptoglobin levels, suggests an increase in plasma protein oxidation.^22^ Hokka et al. further corroborated this association in a prospective observational study of 74 adult patients undergoing cardiovascular surgery requiring CPB. Their multivariate analysis revealed that minimal perioperative haptoglobin levels independently predicted postoperative AKI risk (OR = 0.95, 95 % CI 0.91‒1.0, *p* = 0.03).^16^ Most significantly, Kubota's retrospective study translated these findings by evaluating therapeutic haptoglobin administration during cardiac surgery, providing the first clinical evidence that haptoglobin supplementation may modify AKI risk. After propensity score matching, the incidence of AKI was significantly lower in the group treated with haptoglobin compared to those untreated (OR = 0.65, 95 % CI 0.43‒0.97, *p* = 0.033). These findings support the proposed pathophysiological mechanism whereby hemoglobinemia during cardiac surgery depletes endogenous haptoglobin reserves and suggest that exogenous haptoglobin supplementation may effectively mitigate hemoglobin-mediated nephrotoxicity during surgery.^23^

Additionally, similar findings have been observed in other patient populations. In a retrospective study of 130 critically burned patients, multivariate analysis revealed that undetectable haptoglobin was associated with AKI (OR = 8.32, 95 % CI 2.86‒26.40, *p* < 0.001).^18^ Similarly, in Acute Respiratory Distress Syndrome (ARDS) patients receiving Venovenous Extracorporeal Membrane Oxygenation (VV-ECMO), Graw et al. demonstrated that AKI development correlated significantly with lower plasma haptoglobin levels among those with elevated free hemoglobin.^17^ Likewise, Greite et al. conducted a prospective study and discovered that patients who experienced AKI following Lung Transplantation (LuTx) had significantly reduced haptoglobin levels at the conclusion of surgery compared to those who did not experience AKI.^24^ This phenomenon can also be observed in the present study, where early lower levels of haptoglobin in sepsis patients were associated with a higher incidence of AKI. After comprehensive adjustment for potential confounders, the inverse association remained stable, establishing haptoglobin as an independent protective factor against AKI in sepsis.

However, Wetz et al. observed a moderate negative correlation between free hemoglobin and haptoglobin concentrations during cardiac surgery, but found no significant differences in perioperative haptoglobin concentration between patients with and without AKI occurrence, leading to the conclusion that there is no association between haptoglobin and AKI.^25^ Compared with the present study, Wetz et al. focused on a distinct patient population of elective cardiac surgery patients, while the authors examined sepsis patients, who demonstrate fundamentally different pathophysiology regarding hemolysis and oxidative stress responses. Additionally, with a sample size nearly 9 times larger than theirs (*n* = 1326 vs. 154), this study had significantly enhanced power to detect this association. Most critically, their analysis did not adjust for potential confounders such as blood pressure, SOFA scores, or baseline renal function, which may have obscured the true relationship.

Currently, the mechanisms by which haptoglobin reduces AKI in sepsis patients are not well understood. Studies show that sepsis has a broad impact on Red Blood Cells (RBC), including increased distribution width, increased rigidity as indicated by decreased deformability, and a reduction in RBC membrane proteins.^26^ Additionally, there is an interaction between endotoxins and RBC membranes, leading to damage to the RBC membrane or triggering hemolysis.^27^ The destruction of RBCs results in a rapid increase in circulating cell-free hemoglobin. Haptoglobin, due to its ability to quickly capture and bind free hemoglobin, may mitigate the renal injury caused by oxidative stress and inflammatory responses associated with free hemoglobin during sepsis.^6^ At present, the timing of haptoglobin treatment, pharmacokinetics, and pharmacodynamics assessments are also important research directions.^12^^,^^28^ More clinical studies are needed to clarify the appropriate range of haptoglobin levels in sepsis patients to provide a reference for treatment.

The limitations of this study should be acknowledged. Firstly, the observational design of the study confines the present analysis to the correlation between haptoglobin levels and Acute Kidney Injury (AKI), rather than determining causality. Secondly, as a single-center study with a predominantly American population, the generalizability of these results may be constrained. Thirdly, the absence of free hemoglobin data in this database precludes us from assessing whether sepsis patients may experience haptoglobin depletion due to elevated levels of free hemoglobin, and whether sufficient levels of haptoglobin could counteract the renal injury induced by free hemoglobin. Fourthly, while the authors controlled for numerous confounding factors, there remains the possibility of unmeasured confounders that could affect the study's conclusions. Considering these limitations, future studies using a prospective, multicenter cohort design are essential to validate these findings.

## Conclusion

In conclusion, the present study demonstrates that elevated early haptoglobin levels in sepsis patients are independently linked to a lower risk of AKI. These findings suggest haptoglobin could serve as both a biomarker for early identification of high-risk patients and a potential therapeutic target for preventing sepsis-associated AKI in clinical practice.

## Ethics statement

In accordance with local legislation and institutional requirements, ethical review and approval were not required for the study involving human participants. The studies involving human participants have been reviewed and approved by Beth Israel Deaconess Medical Center. To protect patient privacy, all data were de-identified.

## Funding

The author(s) declare that no financial support was received for the research, authorship, and/or publication of this article.

## CRediT authorship contribution statement

**Yue Liao:** Conceptualization, Resources, Investigation, Methodology, Data curation, Writing – original draft. **Qilin Yang:** Conceptualization, Methodology, Software, Validation, Formal analysis. **Daxue Li:** Methodology, Software, Validation, Formal analysis. **Yulong Wang:** Conceptualization, Methodology, Investigation, Supervision, Project administration, Writing – review & editing.

## Declaration of competing interest

The authors declare no conflicts of interest.

## Appendix Supplementary materials

Image, application 1

Image, application 2

## References

[1] Singer M., Deutschman C.S., Seymour C.W., Shankar-Hari M., Annane D., Bauer M. (2016). The third international consensus definitions for sepsis and septic shock (sepsis-3). JAMA.

[2] Rudd K.E., Johnson S.C., Agesa K.M., Shackelford K.A., Tsoi D., Kievlan D.R. (2020). Global, regional, and national sepsis incidence and mortality, 1990-2017: analysis for the Global Burden of Disease Study. Lancet (Lond Engl).

[3] Watson R.S., Carrol E.D., Carter M.J., Kissoon N., Ranjit S., Schlapbach L.J. (2024). The burden and contemporary epidemiology of sepsis in children. Lancet, Child Adolesc Health..

[4] Zarbock A., Nadim M.K., Pickkers P., Gomez H., Bell S., Joannidis M. (2023). Sepsis-associated acute kidney injury: consensus report of the 28th Acute Disease Quality Initiative workgroup. Nat Rev Nephrol.

[5] Kuwabara S., Goggins E., Okusa M.D. (2022). The pathophysiology of sepsis-associated AKI. Clin J Am Society Nephrol: CJASN.

[6] Kerchberger V.E., Ware L.B. (2020). The role of circulating cell-free hemoglobin in sepsis-associated acute kidney injury. Semin Nephrol.

[7] Effenberger-Neidnicht K., Hartmann M. (2018). Mechanisms of hemolysis during sepsis. Inflammation.

[8] Van Avondt K., Nur E., Zeerleder S. (2019). Mechanisms of haemolysis-induced kidney injury. Nat Rev, Nephrol..

[9] di Masi A., De Simone G., Ciaccio C., D’Orso S., Coletta M., Haptoglobin Ascenzi P. (2020). From hemoglobin scavenging to human health. Mol Aspects Med.

[10] Andersen C.B.F., Stødkilde K., Sæderup K.L., Kuhlee A., Raunser S., Graversen J.H. (2017). Haptoglobin. Antioxid Redox Signal.

[11] Vallelian F., Buehler P.W., Schaer D.J. (2022). Hemolysis, free hemoglobin toxicity, and scavenger protein therapeutics. Blood.

[12] Buehler P.W., Humar R., Schaer D.J. (2020). Haptoglobin therapeutics and compartmentalization of cell-free hemoglobin toxicity. Trends Mol Med.

[13] Janz D.R., Bastarache J.A., Sills G., Wickersham N., May A.K., Bernard G.R. (2013). Association between haptoglobin, hemopexin and mortality in adults with sepsis. Crit Care (Lond Engl).

[14] Lan P., Yu P., Ni J., Zhou J. (2022). Higher serum haptoglobin levels were associated with improved outcomes of patients with septic shock. Crit Care (Lond Engl).

[15] Condello I., Morvillo J.B., Fiore F., Teora V., Nasso G., Speziale G. (2024). Hemadsorption to contain postoperative cell-free hemoglobin and haptoglobin preservation for extended cardiopulmonary bypass time in cardiac surgery for acute kidney injuries prevention. Braz J Cardiovasc Surg.

[16] Hokka M., Egi M., Kubota K., Mizobuchi S. (2021). Perioperative serum free hemoglobin and haptoglobin levels in valvular and aortic surgery with cardiopulmonary bypass: their associations with postoperative kidney injury. J Cardiothorac Vasc Anesth.

[17] Graw J.A., Hildebrandt P., Krannich A., Balzer F., Spies C., Francis R.C. (2022). The role of cell-free hemoglobin and haptoglobin in acute kidney injury in critically ill adults with ARDS and therapy with VV ECMO. Crit Care (Lond Engl).

[18] Dépret F., Dunyach C., De Tymowski C., Chaussard M., Bataille A., Ferry A. (2017). Undetectable haptoglobin is associated with major adverse kidney events in critically ill burn patients. Crit Care (Lond Engl).

[19] Johnson A.E.W., Bulgarelli L., Shen L., Gayles A., Shammout A., Horng S. (2023). MIMIC-IV, a freely accessible electronic health record dataset. Sci Data.

[20] von Elm E., Altman D.G., Egger M., Pocock S.J., Gøtzsche P.C., Vandenbroucke J.P. (2007). Strengthening the reporting of Observational Studies in Epidemiology (STROBE) statement: guidelines for reporting observational studies. BMJ (Clinical research ed).

[21] de Boer I.H., Khunti K., Sadusky T., Tuttle K.R., Neumiller J.J., Rhee C.M. (2022). Diabetes management in Chronic Kidney disease: a consensus report by the American Diabetes Association (ADA) and Kidney Disease: improving global outcomes (KDIGO). Diabetes Care.

[22] Mamikonian L.S., Mamo L.B., Smith P.B., Koo J., Lodge A.J., Turi J.L. (2014). Cardiopulmonary bypass is associated with hemolysis and acute kidney injury in neonates, infants, and children*. Pediatr Crit Care Med: J Soc Crit Care Med World Fed Pediatr Intensive Crit Care Soc.

[23] Kubota K., Egi M., Mizobuchi S. (2017). Haptoglobin administration in cardiovascular surgery patients: its association with the risk of postoperative acute kidney injury. Anesth Analg.

[24] Greite R., Wang L., Gohlke L., Schott S., Kreimann K., Doricic J. (2022). Cell-free hemoglobin in acute kidney injury after lung transplantation and experimental renal ischemia/reperfusion. Int J Mol Sci.

[25] Wetz A.J., Richardt E.M., Schotola H., Bauer M., Bräuer A. (2017). Haptoglobin and free haemoglobin during cardiac surgery-is there a link to acute kidney injury?. Anaesth Intensive Care.

[26] Bateman R.M., Sharpe M.D., Singer M., Ellis C.G. (2017). The effect of sepsis on the erythrocyte. Int J Mol Sci.

[27] Brauckmann S., Effenberger-Neidnicht K., de Groot H., Nagel M., Mayer C., Peters J. (2016). Lipopolysaccharide-induced hemolysis: evidence for direct membrane interactions. Sci Rep.

[28] Galea I., Bandyopadhyay S., Bulters D., Humar R., Hugelshofer M., Schaer D.J. (2023). Haptoglobin treatment for aneurysmal subarachnoid hemorrhage: review and expert consensus on clinical translation. Stroke.

