# Supplementary material for: Inverse relationship between serum haptoglobin and acute kidney injury in critically Ill patients with sepsis: A retrospective cohort study of the MIMIC-IV 3.0 database
Source: Clinics (Sao Paulo). 2025 Jul 30;80:100725. doi: 10.1016/j.clinsp.2025.100725 (PMC12335995; doi:10.1016/j.clinsp.2025.100725)
Supplement: Supplementary file 1 [file mmc1.docx]

CLINICS-D-24-01003_Supplementary Material

Table 1 **|** Details of missing values.

| **Variables** | **The number of missing values** | **The percent of missing values(%)** |
| --- | --- | --- |
| Gender | 0 | 0.00 |
| Age | 0 | 0.00 |
| SBP | 0 | 0.00 |
| DBP | 0 | 0.00 |
| Respiratory rate | 0 | 0.00 |
| Temperature | 16 | 1.21 |
| WBC | 1 | 0.08 |
| Platelets | 0 | 0.00 |
| Glucose | 1 | 0.08 |
| ALT | 128 | 9.67 |
| AST | 122 | 9.21 |
| Bilirubin total | 63 | 4.76 |
| BUN | 0 | 0.00 |
| Creatinine | 0 | 0.00 |
| Sodium | 0 | 0.00 |
| Potassium | 0 | 0.00 |
| Chloride | 0 | 0.00 |
| Hypertension | 0 | 0.00 |
| Diabetes | 0 | 0.00 |
| Chronic pulmonary disease | 0 | 0.00 |
| APSIII score | 0 | 0.00 |
| SOFA score | 0 | 0.00 |
